# Therapy-Induced Senescence: An “Old” Friend Becomes the Enemy

**DOI:** 10.3390/cancers12040822

**Published:** 2020-03-29

**Authors:** Tareq Saleh, Sarah Bloukh, Valerie J. Carpenter, Enas Alwohoush, Jomana Bakeer, Sarah Darwish, Belal Azab, David A. Gewirtz

**Affiliations:** 1 Department of Basic Medical Sciences, Faculty of Medicine, The Hashemite University, Zarqa 13133, Jordan; tareq@hu.edu.jo (T.S.); sarahtdarwish@gmail.com (S.D.); 2 Department of Pathology, Microbiology and Forensic Medicine, School of Medicine, The University of Jordan, Amman 11942, Jordan; sbloukh@gmail.com (S.B.); enasamal@gmail.com (E.A.); j.bakeer95@gmail.com (J.B.); azabbm@vcu.edu (B.A.); 3 Department of Pharmacology and Toxicology, School of Medicine, Virginia Commonwealth University, Richmond, VA 23284, USA; delucavj@vcu.edu; 4 Department of Human and Molecular Genetics, School of Medicine, Virginia Commonwealth University, Richmond, VA 23284, USA

**Keywords:** senescence, cancer, cancer therapy, reversibility, dormancy, recurrence, senolytic

## Abstract

For the past two decades, cellular senescence has been recognized as a central component of the tumor cell response to chemotherapy and radiation. Traditionally, this form of senescence, termed Therapy-Induced Senescence (TIS), was linked to extensive nuclear damage precipitated by classical genotoxic chemotherapy. However, a number of other forms of therapy have also been shown to induce senescence in tumor cells independently of direct genomic damage. This review attempts to provide a comprehensive summary of both conventional and targeted anticancer therapeutics that have been shown to induce senescence in vitro and in vivo. Still, the utility of promoting senescence as a therapeutic endpoint remains under debate. Since senescence represents a durable form of growth arrest, it might be argued that senescence is a desirable outcome of cancer therapy. However, accumulating evidence suggesting that cells have the capacity to escape from TIS would support an alternative conclusion, that senescence provides an avenue whereby tumor cells can evade the potentially lethal action of anticancer drugs, allowing the cells to enter a temporary state of dormancy that eventually facilitates disease recurrence, often in a more aggressive state. Furthermore, TIS is now strongly connected to tumor cell remodeling, potentially to tumor dormancy, acquiring more ominous malignant phenotypes and accounts for several untoward adverse effects of cancer therapy. Here, we argue that senescence represents a barrier to effective anticancer treatment, and discuss the emerging efforts to identify and exploit agents with senolytic properties as a strategy for elimination of the persistent residual surviving tumor cell population, with the goal of mitigating the tumor-promoting influence of the senescent cells and to thereby reduce the likelihood of cancer relapse.

## 1. Introduction

The definition of cellular senescence has evolved dramatically in the years since Hayflick and Morehead first observed replicative senescence in the 1960s. Hayflick successfully challenged the prevailing paradigm that cells growing in vitro can divide indefinitely [1]. Through a series of careful experiments, he demonstrated that human fibroblasts are not immortal, but rather enter a senescent phase wherein they are incapable of further division [1]. Hayflick considered senescence to be an “eternal” fate, believing that senescent cells are committed to an irreversible growth arrest [2,3]. This premise for many years provided the foundation for our understanding of senescence. For example, “irreversibility” was long considered a critical characteristic that distinguished senescence from other forms of growth arrest such as quiescence, a transient form of growth arrest [4]. However, over the past few decades, hallmarks of senescence have been identified that collectively characterize a more complex, unique phenotype, that does not simply reflect another variant of growth arrest [5]. This phenotype comprises extensive genetic, epigenetic, metabolic, and structural alterations which further complicate the early views of senescence. Nevertheless, the stable nature of the growth arrest long remained a fixed component in the definition of senescence [6].

A number of biological contributions of cellular senescence in homeostatic and pathological processes have also been identified [7]. For example, the induction of senescence in response to telomere shortening occurring as a consequence of successive cell duplication (i.e., Replicative Senescence, RS) is not only an indicator of cellular mortality and aging but represents a fundamental tumor-suppressor mechanism [8,9]. That is, the stability of senescent growth arrest is a barrier against the progression of genetically unstable cells that carry a dangerous malignant potential, which accounts for the accumulation of senescent cells in premalignant lesions [10]. The tumor-suppressive role of senescence is derived from studies by multiple laboratories that demonstrated the development of senescence in somatic cells in response to oncogene overexpression (Oncogene-Induced Senescence, OIS) [11,12,13,14,15]. This tumor-suppressive trait of senescence is also related to its role as a stress response to noxious stimuli such as oxidative stress, which partially explains the increased burden of senescent cells in aging organisms [16]. In fact, senescence is a pivotal mechanism of cellular aging and its involvement in an array of aging-related pathologies is strongly documented. For instance, senescence has established roles in the pathogenesis of vascular atherosclerosis, pulmonary fibrosis, osteoarthritis, Alzheimer’s disease, obesity, kidney disease and, of course, cancer [17,18,19,20,21,22,23]. In this context, cancer cells, which are, by definition, immortal, can nevertheless undergo senescence in response to severe stress induced by the exposure to a wide variety of cancer therapeutics. This variant of senescence is often termed, Therapy-Induced Senescence (TIS).

The traditional understanding of senescence as an “irreversible” mechanism whereby tumor proliferation can be abrogated for a prolonged period of time would support senescence as a favorable response to cancer therapies [24,25], and the development of senescence-inducing therapies as cancer treatments [26]. However, recent years have seen the accumulation of a critical mass of studies in support of a countervailing conclusion, specifically that senescent cells are not permanently arrested, and can, in fact, potentially resume proliferation and generate tumors both in vitro and in vivo [27]. That is, while the growth-inhibitory consequence of senescence is likely to be initially beneficial, recent evidence has demonstrated that the accumulation of senescent tumor cells could contribute to unfavorable outcomes of conventional cancer therapy, including the emergence of a more malignant phenotype [28]. This review attempts to provide a comprehensive summary of the therapeutics that have been shown to induce senescence in tumor cells, which argues against the utility of senescence as a therapeutic endpoint and summarizes the strategy of utilizing senolytic agents as an anticancer adjuvant therapy.

## 2. Senescence as an Established Response to an Array of Anticancer Chemotherapeutics In Vitro and In Vivo

Although tumor cells are, by definition, immortal, which is in large part due to the re-expression of telomerase [29], it has been well established that senescence unrelated to telomere shortening or dysfunction can be induced by cancer chemotherapeutic drugs and radiation. The first chemotherapeutics shown to induce senescence in tumor cells were genotoxic agents such as cisplatin [30] and doxorubicin [31]. These drugs generate many of the hallmarks of senescence in tumor cells, including enhanced expression of Senescence Associated-β-galactosidase (SA-β-gal), prolonged growth arrest, and polyploidy (reviewed in [32]). Importantly, doxorubicin-induced senescence has also been demonstrated in patient tumor samples, indicating that the ability of tumor cells to undergo senescence in response to therapy is not exclusive to the experimental setting [33]. Therapy-Induced Senescence (TIS) is now an established response to a wide array of chemotherapeutic agents, both conventional and targeted, and has been established as a target for cancer therapy [34]. The list of senescence-inducing agents is summarized in Table 1.

### 2.1. Topoisomerase Poisons/Inhibitors

This class of anticancer drugs is classically divided into two groups, drugs that target either topoisomerase I or topoisomerase II. Inhibition of the ligation function of topoisomerases results in extensive single- and double-stranded DNA damage. Topoisomerase inhibitors are now considered classical inducers of senescence in both tumor and non-tumor cells, and are often used experimentally as positive controls to test the ability of a cell model to undergo senescence. Topoisomerase inhibitors were also demonstrated to induce senescence in vivo, both in animal models of cancer and in cancer patients receiving these drugs as part of their adjuvant or neoadjuvant therapy [33].

Doxorubicin, or Adriamycin, a natural anthracycline, is a widely used chemotherapeutic for the treatment of a variety of solid and soft tissue cancers. It is an essential component of chemotherapeutic drug combinations used against several subtypes of leukemia and is a leading chemotherapeutic for the treatment of breast cancer. Doxorubicin acts primarily via inhibition of topoisomerase II, but also exerts its antitumor effect by generating extensive oxidative stress. Our laboratory as well as others originally reported that doxorubicin induces robust senescence in breast tumor cell lines. Specifically, doxorubicin promotes SA-β-gal expression in MCF7 breast tumor cells indicative of senescence induction, which is largely p53-dependent [35]. Of particular note, we provided early evidence that TIS is not precipitated by telomeric dysfunction as it was shown to be independent of telomere length [35]. Doxorubicin-induced senescence was also shown to be dependent on the activation of the BMP4-Smad pathway and the contribution of two central cell cycle inhibitors: p21^Cip1^ and p16^INK4^ [36]. Despite appearing to be major contributors to the induction of senescence, p53, p21^Cip1^ and p16^INK4^ are not absolutely required for doxorubicin-induced senescence [37], which has been reported in a wide variety of tumor cell lines (reviewed in [32] and [134]), of which we provide a few examples (Table 1). Daunorubicin, another anthracycline used in the treatment of leukemia, has also been reported to induce senescence when used at sublethal concentrations in studies in Jurkat T lymphocytes [44].

Etoposide, a semisynthetic derivative of the plant-derived podophyllotoxin, is commonly used for the treatment of lung, testicular and ovarian cancers as well as lymphoma and leukemia. Etoposide is a topoisomerase II poison and preferentially induces both apoptosis and senescence in tumor cells as a function of the concentration utilized. For example, low concentrations of etoposide induce senescence in HepG2 cells marked by increased SA-β-gal, p53 and p21^Cip1^ expression [45]. Interestingly, it was shown that early temporal dynamics of p53 transcription in response to low dose etoposide dictate the cell’s decision to either undergo apoptosis or senescence [45]. It was further established that p21^Cip1^, activated downstream of p53 in response to etoposide, is essential for maintaining of the viability of senescent cells and prevention of their accelerated decline into cell death, which could explain, in part, the persistence of senescent cells in vivo [46]. In addition to promoting classical features of senescence, etoposide also induces the expression of multiple Senescence-Associated Secretory Phenotype (SASP) factors in several tumor cell lines such as B16F10 melanoma and NRK-52E rat renal tubular epithelial cells [47,48]. Although a literature search has not identified any studies assessing the ability of etoposide to induce senescence in vivo, we have shown that etoposide increases SA-β-gal expression in A549 xenografts in an immunocompromised animal model (unpublished data). Mitoxantrone, another topoisomerase II inhibitor, is also known to induce senescence and SASP in prostate tumor cells both in vitro and in vivo [50], as well as in A549 lung tumor cells in vitro [51].

Topoisomerase I inhibitors have also been shown to induce senescence in tumor cell lines. For example, camptothecin, at relatively low concentrations, induced senescence in HCT116 colon cancer cells [52]. Again, p53 and p21^Cip1^ have an important role in mediating camptothecin-induced senescence in tumor cells, with sustained p21^Cip1^ apparently responsible for the viability of senescent cells harboring extensive DNA damage [52]. In fact, inhibition of autophagy, a metabolic cell stress response often accompanying senescence, in camptothecin-senescent cells, results in their demise, partly due to inhibition of the p53-p21^Cip1^ pathway [53].

The causation of single-stranded DNA breaks during DNA replication appears to be sufficient to induce senescence, indicating that different forms of genotoxicity, i.e., single- or double-stranded breaks, can activate pathways leading to senescence induction [54]. The camptothecin derivative, irinotecan, generated a senescence response in SGC-7901 and MKN-45 GC gastric carcinoma cell lines [56], while repeated administration of low concentrations of another clinically relevant camptothecin derivative, topotecan, induced senescence in MYCN-amplified neuroblastoma cells [55]. Topotecan was also reported to be capable of inducing senescence features in neuroblastoma xenografts in vivo marked by SA-β-gal upregulation and p21^Cip1^ induction [55]. The latter observation provides additional evidence of the ability of cytotoxic therapies to induce senescence in tumor-bearing animal models.

### 2.2. Alkylating Agents

Alkylating agents have been used extensively for the treatment of a variety of soft and solid tissue cancers. These agents can induce severe genotoxic stress by initiating a nucleophilic attack against guanine bases by attaching an alkyl group to nitrogen atom number 7, thereby resulting in DNA cross-linking. Busulfan, an alkylating agent frequently used for the treatment of chronic myelogenous leukemia (CML), has been shown to induce senescence in several tumor and non-tumor cell lines, including mesenchymal stem cells, fibroblasts and U2OS and MG63 human osteosarcoma cells [42,57,58]. Senescence induction by busulfan in fibroblasts was attributed to the activation of the Erk-p38MAPK pathway in response to oxidative stress [57,59]. Despite its primary use against liquid malignancies, there is little or no preclinical evidence for busulfan inducing senescence in leukemia cells; however, busulfan has been shown to induce accelerated senescence in murine bone marrow cells, which could potentially explain its hematopoietic adverse effects [60].

Temozolomide is an oral alkylating agent that has been a cornerstone in the treatment of brain tumors such as astrocytomas and glioblastoma multiforme. Temozolomide is capable of inducing features of senescence in several immortalized human, murine and patient-derived glioma cell lines [61,62]. Temozolomide induces senescence by introducing the O^6^MeG lesion, which results in the activation of the DNA Damage Repair Response (DDR) ATR-CHK1 pathway, eventually forcing a p21^Cip1^-mediated growth arrest [63]. Moreover, temozolomide resulted in the accumulation of p16^INK4^ positive, senescent cells in mice, demonstrating its senescence-inducing potential in vivo [28].

Other alkylating agents, such as carmustine, dacarbazine, cyclophosphamide, and melphalan have also been shown to induce senescence in a variety of cell lines [62,64]. Dacarbazine, in combination with cisplatin, induced robust SASP in A375 and B16F10 cell lines, despite the absence of significant SA-β-gal expression [64]. Moreover, both melphalan and cyclophosphamide were able to induce senescence in multiple myeloma and lymphoma mouse models, respectively [65,66].

### 2.3. Platinum-Based Drugs

Platinum-based compounds represent a major class of antineoplastic drugs that are used for the treatment of most solid tumors. Platinum-based drugs induce extensive DNA damage in the form of intra- and interstrand crosslinks as well as DNA-protein crosslinking, and are first-line treatments for lung, ovarian and testicular cancers. Cisplatin was the first compound tested for the induction of TIS in tumor cells. Cisplatin induces senescence in several melanoma cell lines associated with a vigorous secretory response [64]. Cisplatin also induces senescence in A2780, SKOV3 and TOV-21G ovarian cancer cells; A549 and H292 lung cancer cells; HepG2 and SMMC-7721 hepatocellular cancer cells and CNE1 nasopharyngeal cancer cells [30,68,69,70]. In addition, carboplatin, a more tolerable cisplatin analogue, was shown to induce senescence in tumor samples obtained from non-small cell lung cancer patients who received carboplatin + docetaxel neoadjuvant therapy [71]. Finally, oxaliplatin is also capable of activating senescence in tumor cells of rats harboring large colorectal tumors [72].

### 2.4. Antimetabolites

Antimetabolites represent one of the earliest classes of antitumor drugs. Drugs of this class exert their antitumor effects by interfering with essential metabolic pathways required for nucleic acid synthesis. For example, methotrexate, an antimetabolite commonly used for the treatment of leukemia, lymphoma and breast cancer, inhibits the enzyme dihydrofolate reductase, which is responsible for generating tetrahydrofolate species necessary for DNA synthesis. Methotrexate induced accelerated senescence in C85 colon cancer cells [74,75]. Methotrexate-induced senescence was primarily p53-dependent and associated with vast genetic alterations permissive for the tumor cell to acquire inflammatory (secretory) and migratory characteristics [74,75]. In support of a role for p53 in mediating methotrexate-induced senescence, we had shown that MCF-7 breast tumor cells with attenuated p53 function were growth-arrested in response to methotrexate exposure, but did not express other markers associated with the senescent phenotype [76]. Pemetrexed, another important antifolate used for the treatment of blood, breast and lung malignancies, is also capable of inducing senescence and SASP in a variety of cancer cell lines in vitro [77,78].

Gemcitabine, a nucleoside analogue used primarily for the treatment of pancreatic cancer, can generate oxidative stress sufficient to activate senescence in Miapaca-2 and Panc-1 human pancreatic cancer cells marked by growth arrest and SA-β-gal expression [79]. Azacitidine, a pyrimidine analogue used for myelodysplastic syndrome, induces senescence in a variety of cell lines in vitro including U2OS human osteosarcoma, MCF7 breast cancer, TPC-1 papillary thyroid carcinoma, DU145 and LNCaP prostate cancer, and several cholangiocarcinoma cell lines [40,81,82,83]. Similarly, bromodeoxyuridine induced senescence in vitro in several tumor cell lines [55,83]. 5-Fluorouracil, another pyrimidine analogue widely used for the treatment of gastrointestinal, breast and skin cancers, can also induce a senescent response in SMMC-7721 hepatocellular cancer and triple-negative MDA-MB-231 breast cancer cells [84,85].

A related immunosuppressant, mycophenolic acid, a potent inosine monophosphate dehydrogenase (IMPDH) inhibitor, was shown to induce senescence in imatinib-resistant chronic myelogenous leukemia cells [86], while hydroxyurea induced senescence in MYCN-amplified neuroblastoma cells [87].

### 2.5. Microtubule Inhibitors

Microtubule poisons are effective in activating apoptosis or mitotic catastrophe in tumor cells, but are generally considered to be relatively weak in inducing senescence [31]. However, paclitaxel, a potent microtubule inhibitor that stabilizes the mitotic spindle and interferes with its polymerization, is known to induce senescence in MCF7 breast tumor cells [89]. Furthermore, paclitaxel has been shown to induce senescence in other non-tumor cell types [28,113]. Docetaxel, on the other hand, induces senescence in prostate cancer models [40,90]. Vinca alkaloids, such as vincristine and vinblastine, are widely used for the treatment of blood malignancies. Vincristine, which is a mitotic spindle destabilizer, induces senescence in MCF7 breast tumor cells, while vinblastine has been shown to amplify temozolomide’s senescence-inducing potential in resistant glioma cells [61,91].

### 2.6. Hormonal Therapy

For breast cancer patients that are estrogen receptor positive (ER+), the predominant hormonal therapies are antiestrogens/selective estrogen receptor modulators (SERMs) and aromatase inhibitors. SERMs block the action of estrogen at the receptor level, while aromatase inhibitors limit the production of estrogen. However, there is relatively limited literature on the capacity of SERMs to induce senescence. Fulvestrant and tamoxifen have both been shown to induce senescent phenotypes in breast cancer cell lines, but the percentage of senescence induced in the population is typically less than 50% [92,94,95]. One study using the charcoal-stripped-serum (CSS) model, which removes all steroid hormones from the medium, demonstrated a more robust senescent phenotype in MCF-7 cells in response to estrogen deprivation [93]. While these studies are limited, they do suggest that estrogen/estrogen receptor modulation can lead to a moderate senescence response in ER+ cancer cells. To our knowledge, there is no evidence for or against the induction of senescence by aromatase inhibitors. With regard to prostate cancer, the standard-of-care therapies are luteinizing-hormone-releasing-hormone (LHRH) receptor agonists or antagonists and antiandrogens. LHRH therapies limit the testicular production of testosterone, while antiandrogens antagonize the androgen receptor directly. In vitro, the effects of LHRH therapies are modeled by using CSS, similar to some studies in breast cancer. The CSS model (and in a few studies, the antiandrogens bicalutamide and enzalutamide) has been shown to induce senescence in prostate cancer cells [96,97,98,99]. Notably, the Jarrard group has also demonstrated that androgen deprivation may induce senescence in vivo as well, in both castrated tumor-bearing mice and treated patient biopsy samples [99].

### 2.7. Kinase Inhibitors

Protein kinase inhibitors are amongst the early targeted therapies developed for cancer treatment. These drugs target a diversity of biochemical pathways implicated in cancer cell growth and survival. Imatinib and nilotinib are small molecule kinase inhibitors that selectively inhibit the kinase activity of the fusion protein BCR-ABL, which is implicated in driving accelerated proliferation in multiple leukemia subtypes such as chronic myelogenous leukemia. Imatinib induces senescence in K562 leukemic cells marked by growth arrest, p21^Cip1^, p27 induction, and enhancement of SA-β-gal activity, while nilotinib induces senescence in H1975 NSCLC cells marked by increased SA-β-gal activity [100,101]. The MEK-1/2 and B-RAF inhibitors trametinib and vemurafenib, respectively, are both approved for use in metastatic V600E mutated melanoma, and both agents have been shown to induce senescence in vitro and in vivo [102,103,104,105,106]. Dasatinib, on the other hand, blocks several tyrosine kinases and promotes p21^Cip1^-dependent senescence by the induction of DNA damage in BRAF-mutant NSCLC cells [107,108].

Inhibition of protein kinase signaling downstream of the HER2 receptor has proven effective in the treatment of HER2-positive breast cancer. A recent report revealed that several of these kinase inhibitors such as lapatinib, neratinib and afatinib induced senescence in HER2-positive breast cancer cells lines marked by robust SA-β-gal induction and growth inhibition [109]. Interestingly, in the tested models, direct HER2 inhibition by trastuzumab monotherapy failed to induce senescence [109]. Conversely, EGFR inhibitors such as gefitinib and erlotinib have been reported to induce senescence in EGFR-mutant and non-mutant NSCLC cell lines [110,111].

### 2.8. mTOR Inhibitors

The mammalian Target of Rapamycin (mTOR) is a fundamental regulator of senescence and aging. Functional mTOR is a component of the active complexes (mTORC1 and mTORC2) and plays a pivotal role in regulating signaling pathways implicated in energy regulation, proliferation and ribosomal protein synthesis [135]. mTORC1 is a known regulator of the SASP and several reports have elucidated its role in promoting the secretion of an array of inflammatory cytokines and chemokines by senescent cells [136]. More importantly, inhibition of mTORC1 by rapamycin was shown to mitigate the inflammatory potential of senescent cells; since the SASP is a primary driver of aging mechanisms precipitated by the accumulation of senescent cells, rapamycin has been used to prolong lifespan and delay certain aging-associated pathologies [137].

Accordingly, mTOR inhibitors are expected to interfere with senescence induction or, at least, the generation of some of its functional indicators. In fact, this outcome has been reported both in vitro, in WI38 fibroblasts, marked by reversal of senescent growth arrest and attenuation of senescence-associated hallmarks, and in vivo, marked by the decreased accumulation of senescent cells in mice [138]. However, we have identified other observations in cancer models where mTOR inhibitors can paradoxically induce senescence. For example, rapamycin (sirolimus) exerted a synergistic, senescence-inducing effect when combined with the antimetabolite, 5-FU, against the SMMC-7721 hepatocellular carcinoma cell line [84]. It is important to note that, in this work, rapamycin alone did not increase the number of SA-β-gal positive cells, but only in combination with 5-FU. Moreover, it was not identified whether or not this rapamycin effect was mediated through mTORC1 inhibition. In another report, both rapamycin and everolimus were able to independently induce senescence, marked by SA-β-gal staining, in HUVEC cells in vitro [113]. In this work, both agents produced a significant decrease in Sirt1 expression, which has a negative regulatory effect on mTOR [139]. Moreover, Sirt1 overexpression was successful in reversing certain features of rapamycin and everolimus-induced senescence [113]. These observations highlight a quite complex role of mTOR in the regulation of senescence.

### 2.9. Monoclonal Antibodies

A number of antibodies that have been developed for the treatment of cancer have been reported to induce senescence in cancer models. These drugs are designed to target particular signaling pathways or molecular targets representing essential cancer hallmarks. For example, both rituximab and obinutuzumab (anti-CD20 monoclonal antibodies) can effectively induce senescence (as well as apoptosis) in follicular lymphoma models [49]. Pertuzumab and trastuzumab, monoclonal antibodies used for the treatment of HER-2-positive breast cancer, were shown to promote senescence in breast cancer models [115]; however, in another report, trastuzumab alone failed to induce robust senescence [109].

Bevacizumab, a monoclonal antibody that targets VEGFA, and thus, interferes with angiogenesis, was approved for the treatment of metastatic colorectal and breast cancers. Inhibition of VEGF binding to its receptor in colorectal cancer cells by bevacizumab was associated with senescence induction [116]. Ranibizumab is another monoclonal antibody fragment antiangiogenic agent that can also induce senescence in porcine retinal pigment epithelial cells [117].

### 2.10. CDK 4/6 Inhibitors

Transition through phases of the cell cycle is facilitated by the activation of different cyclins and Cyclin-Dependent Kinases (CDKs). CDK4 and CDK6 are key drivers of the transition from the G1 to S phases of the cell cycle, primarily through the phosphorylation and consequent inactivation of Rb. In senescence, upregulation of CDKIs such as p21^Cip1^ and p16^INK4^ inhibit the activity of CDKs and thus permit Rb to enforce a stable growth arrest that halts progression through the cell cycle. Several compounds were recently developed to interfere with CDKs, and consequently induce growth arrest in tumor cells. CDK inhibitors have also been shown to act as inducers of senescence.

Palbociclib, a selective CDK4/6 inhibitor and the first in this class drug to be approved for the treatment of ER+/HER-negative breast cancer, is now an established inducer of senescence in tumor cells. The first evidence of palbociclib-induced senescence was generated in 16 different Rb-proficient glioblastoma multiforme tumor cell models. Senescence was confirmed by increased SA-β-gal staining and reduced proliferation (decreased BrdU incorporation) [118]. CDK inhibition was associated with inactivation of FOXM1, which decreased the expression of multiple G1 to S phase transition genes, and thus, induced senescence [119]. Moreover, it was shown that degradation of MDM2 in response to palbociclib exposure was critical for the induction of senescence hallmarks, since the persistence of MDM2 resulted in palbociclib-exposed liposarcoma cells entering into a quiescent, rather than a senescent, growth arrest [120]. It is also noted that palbociclib-induced senescence results from significant inhibition of TORC1 signaling in melanoma cells [121]. However, it must be emphasized here that, despite inducing senescence rapidly in tumor cells, palbociclib-induced senescence appears to be relatively unstable, with senescent tumor cells recovering the potential to proliferate [112]. Interestingly, palbociclib-induced senescence is DNA damage-independent, which might explain why the majority of cells can escape arrest quickly, rather than a few subclones escaping slowly [122]. Lastly, palbociclib was also shown to induce senescence in SK-MEL-103 melanoma xenografts in vivo [123]. Similarly, the CDK4/6 inhibitors, abemaciclib and ribociclib, also induced senescence in ER+ breast cancer and ovarian cancer cell models, respectively [128,129]. CDK inhibitors are reliable senescence inducers and are now more frequently being used for preclinical studies of TIS.

### 2.11. Aurora Kinase Inhibitors

These novel compounds are being investigated as potential anticancer drugs. Aurora kinases regulate progression through the cell cycle, and their inhibition was found to interfere with tumor progression in a number of preclinical studies. Interestingly, it has also been reported that aurora kinase inhibitors also promote senescence in tumor and non-tumor cell lines. For example, MLN8054, a selective Aurora A kinase inhibitor, induces senescence in vitro (enhanced SA-β-gal, senescence morphology, upregulation of p21^Cip1^ and consequent inactivation of Rb) and in vivo in HCT116 models [140]. Interestingly, Aurora A kinase inhibition resulted in activation of the DDR and NF-κB-mediated activation of SASP, a pattern frequently observed with senescence induction by conventional cytotoxic chemotherapy [141]. TAK901, a selective aurora B kinase inhibitor also induces SA-β-Gal upregulation in HCT116 cells [142]. Global Aurora kinases inhibition using AMG900 in combination with HDAC inhibitors resulted in upregulation of senescence features in prostate cancer cell lines in vitro and in vivo [143]. Induction of senescence by Aurora kinase inhibition was associated with activation of cancer immunosurveillance mechanisms and increased clearance of tumor cells [144]. Lastly, a critical report by Wang et al. demonstrated the ability of multiple Aurora kinase inhibitors to induce senescence in a variety of tumor cell lines, confirming their reliability in inducing senescence in preclinical cancer models [145].

### 2.12. PARP Inhibitors

Several PARP inhibitors have recently been approved for the treatment of cancer, particularly, BRCA-mutant breast and ovarian cancers. Inhibition of PARP results in the persistence of DNA single-stranded breaks, which then progress into double-stranded breaks in tumor cells with mutated BRCA1 and BRCA2 (impaired homologous DNA recombination). Accumulation of these DNA lesions frequently commits cells into cell death. However, many preclinical reports have demonstrated that exposure to PARP inhibitors can also result in senescence induction in a variety of tumor models. For example, we have reported that PARP inhibition (by olaparib and niraparib) sensitized tumor cells that have been exposed to ionizing radiation by producing a more robust senescent response associated with increased DNA double-stranded damage [130]. These results were recently confirmed by showing that olaparib, the first FDA-approved PARP inhibitor, results in a robust senescent response (rather than apoptosis) when preceded by genotoxic stress induced by cisplatin or pemetrexed [146].

Similar outcomes were reported upon a combination of low-dose radiation with rucaparib in studies with prostate cancer cells [132]. Rucaparib increased the accumulation of γH2AX-positive DNA double-stranded breaks induced by low-dose radiation, which manifested as a significant increase in SA-β-gal-positive tumor cells [132]. Interestingly, similar to many observations with CDK inhibitors-induced senescence, senescence induced by PARP inhibition (often in combination with other DNA damaging agents) is associated with a relatively unstable cell cycle arrest, where senescent tumor cells can recover reproductive potential [131,147,148].

## 3. Unfavorable Outcomes of Therapy-Induced Senescence and Its Contribution to Cancer Recurrence

The debate about the duration of senescence has long suffered from the circular argument that even if cells expressing essentially all of the hallmarks of senescence are observed to recover from a prolonged state of growth arrest, then the recovering cells could not have originated from the senescent population because senescence is, by definition, irreversible. Consequently, researchers have often resorted to terminology such as “senescence-like” and “pseudo-senescence” in reference to cells with characteristics of senescence that have escaped from prolonged growth arrest [149,150,151]. While this terminology is acceptable, it tends to minimize the validity of the observations that cells exhibiting the hallmarks of senescence can overcome the growth arrested state.

It is key to this argument to emphasize that the “reversibility” of senescence does not reflect an actual reversion to the pre-senescence phenotype analogous to a reversible chemical reaction; rather, escape of tumor cells from senescence appears to be associated with dynamic genotypic and phenotypic changes that lead to the emergence of more malignant processes [152]. It is equally important to note that escape from senescence is likely to be a relatively infrequent event occurring only in certain biological settings, such as the evasion of oncogene-induced senescence during transformation.

Finally, it is essential to understand that the definition of senescence, particularly in the context of genomically-unstable cancer cells, remains somewhat fluid, given newly arising evidence highlighting the considerable transcriptomic heterogeneity of senescent cells [153]. It is further recognized that the SASP possesses a highly dynamic, but also variable profile that is dependent on cell type, duration of senescence induction, and nature of the senescence-inducing stimulus [154]. In this context, it should be emphasized that the literature discussed in the subsequent section on reversion from cell cycle arrest reflect studies that were performed predominantly in in vitro models, where the use and interpretation of senescence markers is relatively reliable. Nevertheless, a recent report by Bojko et al., highlights the limitations of these markers and how even the presentation of TIS in vitro can be highly diverse [43]. Our current ability to capture and to quantify senescence in vivo is limited and the identification of reproducible, reliable and validated markers of senescence in patient tissues would be critical for establishing the extent to which senescence contributes to either the effectiveness of therapy, in terms of a durable growth arrest, and/or the capacity of senescent tumor cells to ultimately recover and re-emerge as a recurrent (and possibly more aggressive) form of disease [155].

### 3.1. Evidence for the Reversibility of Therapy-Induced Senescence (TIS)

Early studies from our group demonstrated that clinically relevant concentrations of Adriamycin induce p53-dependent cellular senescence in MCF7 breast tumor cells [156]. In this study, a small population of MCF7 cells recovered from acute Adriamycin exposure and a senescence-resistant colony was isolated that failed to undergo senescence in response to subsequent exposure to Adriamycin [156]. In comparison with parental cells exposed to Adriamycin, the senescence-resistant colony expressed higher levels of proliferative cell cycle regulators, such as cyclin B1 and E2, PCNA and cdc2, following Adriamycin treatment. In fact, continuous expression of cdc2 is likely to account for the ability of these cells to evade senescence, as also shown in similar studies by Roberson et al., published at the same time as this work [71]. Interestingly, this resistance to induction of senescence by Adriamycin was not due to decreased intracellular drug accumulation or an attenuated DDR since the resistant colony continued to respond to Adriamycin by p53 and p21^Cip1^ induction [156]. These initial experiments suggested that certain tumor cells with altered genetic background could potentially evade senescence or at least acquire senescence-resistant traits.

A number of reports by the Wu group at the University of Washington have largely supported the ability of tumor cells to evade therapy-induced senescence [71,150,157]. In one study, H1299 non-small cell lung cancer (NSCLC) cells, which are null in p53 and deficient in p16^INK4^, were induced into senescence by camptothecin and other chemotherapeutic agents such as etoposide and cisplatin [71]. Since the choice of a cell line lacking pivotal senescence regulatory proteins could be a deficiency, the authors indicated that genes encoding for p53 and p16^INK4^ are frequently mutated in patient malignancies and that consequently this experimental model is reflective of clinical cancer. In this work, senescence induction in the H1299 cells was confirmed based on their large, flat morphology, increased granularity, retention of PKH2 (a cell membrane staining dyeـــ retention indicative of lack of cell division) and finally, upregulated SA-β-gal expression. Interestingly, a small population of senescent cells were subsequently observed to regain proliferative capacity, resulting in the formation of colonies 3–4 weeks after removal of the drug; the frequency of escape was 1 of every 10^6^ cells, suggesting that senescent growth is stable and its evasion is an infrequent occurrence [71]. In contrast to the findings of the Elmore et al. study, where the senescence-resistant cells developed cross-resistance to multiple senescence-inducing drugs [156], the senescence-escaped cell colonies were found to have similar sensitivity to camptothecin as the parental H1299 cells, indicating that these cells did not arise from a senescence-resistant H1299 cell population [71]. However, this escape from senescence was also associated with sustained expression of cdc2, suggesting that cdc2 plays an important role in evasion of senescence. Furthermore, cdc2 levels were elevated in clinical tumor samples from patients who received adjuvant chemotherapy and underwent senescence as marked by SA-β-gal staining [71].

Polyploidy, which reflects cells containing more than three times the number of haploid chromosomes, is a common feature of senescent cells. In a follow up study by the Wu group, Wang et al. demonstrated that many camptothecin-induced senescent H1299 cells are polyploid [150]. Interestingly, a considerable number of polyploid senescent tumor cells were able to show markers of cell proliferation and DNA synthesis. Using live cell imaging and H2B-RFP labeling (that allowed fluorescence visualization of the nucleus), Wang et al. also showed polyploid senescent cells escaping from senescence. To confirm the live imaging study, camptothecin-induced senescent cells were sorted based on size, and the large, polyploid cells were reseeded. Colonies were observed 7–10 days after seeding [150]. Again, escape from senescence was found to be related to overexpression of cdc2 in agreement with the previous observations [35,156]. It was further demonstrated that therapy-induced cell senescence in patients with locally advanced NSCLC was associated with poor prognosis. Wang et al. found that survivin, which is downstream of cdc2, is upregulated and plays a role in escape from senescence, as well as the subsequent viability of the escaped cells [157].

Studies by Mosienak et al. identified polyploidy in HCT116 cells where senescence was again induced by doxorubicin [39]. When polyploidy was abrogated, the cells were unable to escape from senescence. Moreover, MCF-7 cells that were shown to undergo senescence but not polyploidy upon doxorubicin treatment were unable to escape from senescence [39].

Was et al. also established the reversibility of senescence in their study where HCT116 cells were treated with three cycles of 24 h doxorubicin and 72 h drug-free treatment [158]. Senescence was evident after 13 days based on flattened cell morphology, cell enlargement and increased granularity, SA-β-gal expression, as well as secretion of VEGF and IL8, components of the SASP [158]. Here, as well, senescent cells were shown to exhibit polyploidy. A six-fold increase in cell number 2 weeks following removal of doxorubicin indicated an escape from senescence. Time-lapse imaging revealed proliferating cells in the polyploid, senescent cell population. Consistent with reports in literature of senescent cells exhibiting stem cell features [159,160], CD24+ (about 1.5% of cells) and NANOG exhibiting stem cell-like cells were also observed in the treated cell population [158].

Doin et al. demonstrated that cisplatin treatment both in vivo and in vitro results in an initial cessation of cell division and proliferation that is eventually succeeded by cells recovering from growth arrest [161]. Cisplatin treatment of PROb cells in vitro resulted in significant cell growth arrest but continued DNA replication. Again, live cell imaging using the fluorescent dye, H2B-GFP, showed the emergence of largely senescent giant polyploid cells, which eventually disappeared due to depolyploidization and resumption of cell proliferation through atypical mitotic division [161]. The observation of colonies of cells arising from the senescent cells lends further credence to the capability of senescent cells to generate proliferating progeny. When cisplatin was administered to PROb colon cancer tumor-bearing BD-IX rats, there was significant tumor shrinkage characterized by giant polyploid cells, most of which stained positive for SA-β-gal [161]. Tumors were observed to resume growth 1 month after drug administration during which time SA-β-gal was significantly absent, indicating that cisplatin treatment delayed but did not stop tumor growth [161].

The development of polyploidy followed by depolyploidization and atypical mitotic division was also demonstrated by Rohnalter et al. [162]. In this study, carboplatin-treated SKOV3 ovarian cancer cells proceed through a series of stages that result in a chemo-resistant phenotype [162]. The multi-staged process included mitotic catastrophe, polyploidization, cell cycle arrest, and senescence followed by the acquisition of stemness, increased cell divisions, depolyploidization, and eventually chemoresistance at 21 weeks following treatment. One of the most distinctive features of this multi-staged process was the presence of giant cells that exhibit polyploidy. The process was accompanied by an initial increase in p21^Cip1^ that peaked at day 8 and began to decrease as cell division increased and giant cell numbers decreased [162]. The emergence of a subpopulation of cells that are capable of cell division and are chemo-resistant is supportive of the capability of carboplatin-induced senescent cells to re-emerge from senescence and the possible contribution of senescence to tumor resurgence or treatment relapse.

Sabisz and Sklandanowski studied senescence and escape of a subpopulation of A549 NSCLC cells [149]. Here, senescence was induced with the topoisomerase II inhibitor etoposide. Similar to the Wang et al. study, about 20% of the senescent cells were found to exhibit polyploidy over the course of the study. Levels of p53 and p21^Cip1^ were also found to increase progressively while Cdc2 and cyclin B1 increased after 1–2 days and decreased beyond detection at day 5 [149]. Following drug treatment for 5 days, cells were incubated in drug-free media and between 1% and 3% of the cell population was observed to regain proliferative capacity at 10 days post-treatment. Sabisz and Sklandanowski determined that about 1.1% of the treated cells expressed stem-cell markers (CD34 and CD117); given that this number was very similar to the senescence-escaped cell population, it was suggested that the two cell fractions could be the same, and that cells escaping senescence acquire stem cell-like characteristics [149].

A study by Achutan et al. involving multiple breast cancer cell lines (MCF-7, MDA MB231, and T47D) and primary tumors also revealed that cells that escaped from doxorubicin-induced senescence could be derived from the cancer stem cell population [151]. The small population of cells that escaped senescence was found to exhibit stem cell characteristics and to express increased levels of the stem cell marker CD133 [151].

The cornerstone of these studies may be a recent report by the Clemens Schmitt group [163]. In agreement with previous observations, Milanovic et al. demonstrated that Eμ-Myc-Bcl2-overexpressing lymphoma cells treated with Adriamycin developed both senescence (SA-β-gal staining) and stem cell-related markers (enhanced aldehyde dehydrogenase) [163]. These stem cell-like characteristics were not significantly apparent in the same cells that were treated with Adriamycin but failed to undergo senescence. The authors utilized an inducible expression model for p53 and Suv39h1, which both play a critical role in enforcing the senescent growth arrest. Suv39h1 is a histone methyltransferase responsible for generating the senescence-associated heterochromatic signature, H3K9Me3, which contributes to the repression of certain proliferative genes. Thus, failure to enforce these epigenetic changes can disrupt the transition to senescence. Accordingly, it was feasible to disable these pro-senescence drivers and promote cell cycle progression following Adriamycin-induced senescence. Although the escape from senescence was not fully spontaneous, the authors concluded that p53 inactivation is a common aberration in tumor cells and contributes to the possibility of evading the senescence-mediated cell cycle arrest. Moreover, the cells that escaped senescence were able to undergo senescence again in response to Adriamycin, indicating that no senescence-resistance phenotypes had developed. An additional interesting finding of this work is that cells that escaped TIS and acquired stem cell properties were more aggressive, forming rapidly growing colonies in vitro and more malignant tumors when implanted in vivo (interestingly, in immunocompetent mice). In summary, TIS results in extensive pro-stemness genetic reprograming that facilitates escape from the growth arrested state and could represent a component of relapsed cancer. Another important conclusion from this work is that tumor cells that escaped senescence are more malignant.

In support of the Milanovic et al. study, Yang et al. also demonstrated that A549 NSCLC cells exposed to Adriamycin (100 nM) for 7 days can resume proliferation after 21 days of drug-free culture [152]. However, unlike the studies in the lymphoma cells where p53 inactivation was necessary for escaping senescence, Yang et al. demonstrated a spontaneous reversion of A549 senescent cells into the proliferative state [152]. The cells that escaped senescence exhibited more invasive and migratory properties. In fact, it has recently been reported that doxorubicin-induced senescent breast tumor cells develop a cannibalistic capacity that allows for the engulfment of adjacent senescent and non-senescent neighbors [164]. This acquisition of a phagocytic capability enables senescent cells to hijack nutrient cargos from other cells, which can then be utilized to prolong their survival, providing an additional explanation for how senescent tumor cells can persist following exposure to anticancer therapy [165].

We have recently provided what we believe is direct evidence for the escape from TIS in tumor cells in vitro and in vivo in work that examined the fate of three tumor cell lines (H460 lung, HCT116 colon and 4T1 breast) induced into senescence by exposure to two topoisomerase II inhibitors, etoposide and doxorubicin [27]. Senescence induction in these cell lines was marked by markers of the senescent phenotype, such as morphological alterations (cell flattening and enlargement), increased SA-β-gal activity, upregulation of p21^Cip1^, increased expression of the senescence surrogate BTG1, and formation of DNA damage and heterochromatic foci [27]. These senescent tumor cells underwent a state of growth arrest, but failed to maintain features of the senescent phenotype indefinitely; consequently, over a period of 7 days (or more), these chemotherapy-exposed tumor cells were able to resume proliferation. The resolution from senescence was accompanied by an attenuation of the expression of select SASP components [27].

In order to confirm that growth resumption was not attributable to non-senescent cells that escape genotoxic stress induced by chemotherapy, enrichment of the senescent H460 and HCT116 cell population was carried out based on the expression of SA-β-gal using fluorescence activated cell sorting, and at different temporal points following senescence induction [27]. Enriched, SA-β-gal-positive tumor cells were shown to resume proliferation, marked by real-time live cell imaging which revealed the ability of a subpopulation of tumor cells to undergo spontaneous mitoses. These outcomes were confirmed using advanced High-Speed Live Cell Interferometry (HSLCI) technology [27].

To further investigate whether senescent tumor cells that escape the growth arrested state can generate viable tumors in vivo, enriched, SA-β-gal-positive H460 cells were subcutaneously injected into NSG mice. The formation of tumors was evident within 14 days of tumor cell implantation [27]. Furthermore, and to examine the role of the immune system in controlling the escape from the senescent growth arrest, enriched, SA-β-gal-positive 4T1 breast tumor cells were implanted in the mammary fat pads of immunocompetent and immunodeficient animals [27]. While tumors were generated in both sets of mice, the tumor formation in immunocompetent BALB/c mice was slower than that in immunocompromised NSG mice, suggesting that the immune system is capable of recognizing and suppressing proliferative capacity of senescent tumor cells [27].

These findings that senescent tumor cells can escape and re-emerge into a proliferative state are not limited to chemotherapeutic drugs. Escape from senescence induced by ionizing radiation has also been reported [130,166,167]. Chitikova et al. described the escape from radiation-induced senescence in apoptosis-resistant E1A+E1B cells [167]. Cells were shown to arrest in the G_2_-M phase of the cell cycle while DNA replication significantly decreased 24 h following exposure to ionizing radiation [167]. Resumption of DNA replication occurred 48 hours after treatment with an attendant polyploid cell accumulation. Polyploid cells were enlarged, flattened and expressed SA-β-gal, consistent with senescence. Cell proliferation following the induction of senescence was observed 7 days post-irradiation, accompanied by the increased expression of stem cell markers NANOG and OCT3/4 [167].

In our own studies with MCF-7 breast tumor cells, proliferative recovery was evident after widespread senescence was induced by exposure to 10 Gy ionizing radiation [167]. We have also demonstrated in our work with DNA repair proficient and deficient HCT116 cells, that escapes from radiation-induced senescence was evident with or without Poly (ADP-ribose) polymerase (PARP) inhibition [130].

In summary, an extensive body of evidence has been generated in support of the contention that tumor cells can, in fact, re-enter the cell cycle after senescence.

### 3.2. Deleterious Effects of the Accumulation of Senescent Cells on Outcomes of Cancer Therapy

In addition to the potential for proliferative recovery and the contribution to disease recurrence, senescence may be an undesirable cell fate due to the multiple adverse events associated with the accumulation of senescent cells. Indeed, the Campisi group published a study in which the elimination of senescent cells following doxorubicin treatment ameliorated multiple toxicities, including bone marrow suppression, inflammation, and even tumor recurrence [28]. These pathological effects of senescence can be attributed to both cell non-autonomous mechanisms such as paracrine signaling through the SASP, as well as cell autonomous mechanisms such as transcriptional reprogramming.

The SASP includes a variety of both soluble and insoluble factors, including chemokines, cytokines, matrix metalloproteases, extracellular matrix components, and other signaling molecules (reviewed in [168]). The development of the SASP allows senescent cells (cancerous or not) to interact with and affect their microenvironment; whether this interaction is beneficial or deleterious to patient outcomes is not yet resolved. However, there is significant evidence that the SASP contributes to disease progression. One mechanism by which this may occur is through the promotion of cellular growth in neighboring non-senescent cells. This has been shown in both melanoma and prostate cancer cells, where tumor cells induced into senescence by cisplatin (melanoma) or doxorubicin (prostate) promoted the increased proliferation of untreated, non-senescent tumor cells [64,169]. Interestingly, while these studies demonstrated a conserved ability of senescent cells to promote growth in vitro, the melanoma study illustrated the same effect in vivo whereas the prostate cancer study did not reflect the findings in cell culture. This likely suggests that this effect of the SASP is tumor-type dependent.

The SASP has also been shown to induce epithelial-to-mesenchymal transition (EMT) in neighboring cells, leading to increased invasiveness and stemness [50,170,171,172]. IL-6 and IL-8 appear to be the main contributors of the SASP to this effect [170]. Angiogenesis may also be accelerated by senescent cells, as the SASP includes proangiogenic factors that induce pathological vessel formation [173]. Finally, SASP factors are highly involved in regulating immune cell infiltration. While there is evidence that the SASP may increase immune surveillance of tumors, specifically through natural killer cells and macrophage recruitment (reviewed in [174]), there is contradictory evidence that the SASP results in immune suppression, dependent upon the context. For example, in prostate cancer, it was found that in PTEN- null cancers, the SASP was highly immunosuppressive due to STAT3 activation, allowing for increased tumor growth [175]. There is also evidence that senescent cells can “reprogram” macrophages to adopt an M2 phenotype, favoring angiogenesis and tumor development [176].

Senescent cells also undergo dramatic transcriptional changes during growth arrest, and consequently may adopt more drug-resistant and aggressive phenotypes. For example, TIS has been repeatedly linked to an increase in stem cell markers (reviewed in [177]). While this may, in part, be caused by the SASP, Milanovic et al., as previously discussed, have successfully shown that increases in stemness can also be cell autonomous occurring as part of senescence-associated transcriptional reprogramming [163]. Senescent cells also develop a pro-survival phenotype, characterized by increased expression of anti-apoptotic proteins such as members of the BCL-2 family and decreased expression of pro-apoptotic proteins such as caspase 3 (reviewed in [178]). These changes in apoptotic regulators may confer resistance to apoptosis-inducing agents, including chemotherapy. In a study in prostate cancer, tumor cells induced into senescence by androgen deprivation (CSS) were less sensitive to docetaxel, bortezomib, and flavopiridol [96]. Studies with senescent fibroblasts have also demonstrated increased resistance to apoptotic agents such as staurosporine and radiation [179,180]. These changes in phenotype occurring during the senescent state may drastically influence chemotherapy effectiveness and patient outcome.

It would be remiss to describe senescent cells as being entirely detrimental. As previously stated, the SASP can recruit immune cells and increase tumor clearance [174]. Furthermore, in some contexts, SASP may produce a senescence-inducing rather than proliferation-inducing bystander effect, which could contribute to increased tumor growth control [181,182,183]. This dual role of senescence as potentially being both beneficial and detrimental has been widely recognized in the aging community and increasingly so in the oncology community. Nevertheless, the evidence summarized here strongly argues that patient outcomes may benefit from the elimination of senescent cells.

## 4. Clearance of Senescent Tumor Cells as A New Approach to Prevent or Delay Cancer Relapse

The accumulation of persistent senescent cells is a pivotal component in the pathogenesis of many chronic ageing-related diseases [184]. Consequently, the clearance of senescent cells is believed to be important for delaying, preventing, alleviating, and slowing down the progression of many of these disease processes [184]. As mentioned above, senescent cells accumulate in vivo in response to a variety of cancer therapeutics. These most probably comprise a combination of both tumor and non-tumor cells. This assumption is the major basis for the proposition that developing treatment modalities to eliminate senescent cells might be a useful complementary anticancer approach. In fact, the selective elimination of senescent cells is a core interest of gerontologists and aging biologists. Several efforts were designed to study the outcome of eliminating senescent cells on certain aging-related pathologies, but the breakthrough came with the development of the INK-ATTAC transgenic mouse model that allowed for the selective killing of p16^INK4^-positive senescent cells in vivo [185]. Upon the removal of the senescent cells from these progeroid mice, several aging-related pathologies were mitigated, which was marked by improved muscle structure and function, physical performance and fat deposition [185]. These results first established that senescence is a primary pathological contributor to several aging-related processes, and second, that the elimination of senescent cells can actually interfere with these processes and yield positive physical outcomes. Despite its tremendous contribution to our understanding of the roles of senescence, the applications of this model are only experimental. Therefore, efforts have recently been directed towards the development of compounds that can selectively eliminate senescent cells without harming their healthy neighbors [186]. These compounds have been termed senolytic agents (Table 2).

### 4.1. Dasatinib + Quercetin

In a seminal paper in 2015, it became clear that increased expression of certain pro-survival signaling networks conferred upon senescent cells the capacity to resist apoptosis [186]. Using a library of short-interfering RNAs (siRNAs) directed against key molecular components that were preferentially upregulated in senescent cells, including ephrins (EFNB1 or 3), PI3Kδ, p21^Cip1^, BCL-xL, or plasminogen-activated inhibitor-2 (PAI-2), and senescent preadipocytes (which often accumulate with aging) were eliminated [186]. Interestingly, this cell-killing effect spared proliferating or differentiated cells [186]. Next, after screening 46 compounds that could interfere with these survival pathways, two drugs, dasatinib and quercetin, were shown to exhibit senolytic potential. Dasatinib is an orally administered small molecule that inhibits multiple tyrosine kinases, including BCR-ABL kinase expressed in certain blood malignancies [220]. Dasatinib also inhibits SRC family kinases (including SRC, LKC, YES, FYN), c-KIT, EPHA2, and platelet derived growth factor receptor β (PDGFRβ) [220]. Dasatinib is approved for the treatment of blood malignancies [220]. Quercetin is a flavonol, abundantly present in natural diets [221]. Due to its antioxidant effects, studies have shown that quercetin has a preventive potential for various diseases, such as osteoporosis, some forms of cancer, and lung and cardiovascular diseases [221,222].

Dasatinib alone eliminated senescent human fat cells, while quercetin alone was more effective against senescent human endothelial cells [186]. The combination (often referred to as D+Q) was effective in killing both cell types in vitro and more effective in eliminating senescent mouse embryonic fibroblasts (MEFs) in vivo than each agent alone [186]. In addition, after irradiation of one leg of wild type mice, one dose of this combination reduced p16^INK4^ expression in muscle fat and the number of SA-β-gal-positive cells in irradiated limb adipose tissue [186]. Lastly, D+Q periodic administered over 10–12 weeks to progeroid Ercc1(−/∆) mice extended their lifespan and resulted in delaying ageing-related features such as osteoporosis and loss of intervertebral disk proteoglycans [186].

Using the bleomycin-induced lung injury idiopathic pulmonary fibrosis (IPF) model, another report proved that the senolytic cocktail D+Q can selectively eliminate senescent lung fibroblasts, thereby ameliorating interstitial remodeling and compromised lung function [18]. The role of senescence in the pathogenesis of IPF is still not fully understood; however, the prompt suicide-gene-based senescent cell clearance evidently enhanced lung function despite the lack of gross improvement in lung fibrosis. Consequently, the D+Q senolytic cocktail has been proposed as a beneficial therapeutic intervention against IPF [18]. This was later confirmed by another report demonstrating the effective senolytic activity of D+Q against senescent alveolar epithelial cell types ex vivo [187]. Additionally, the D+Q combination was effective in ameliorating features of vascular pathology in aging mice and hypercholesterolemia (atherosclerosis) animal models possibly via the selective clearance of senescent cells [188].

Because of these promising effects of D+Q, and other phytochemicals, the combination was proposed as a potential approach to eliminate senescent tumor and non-tumor cells that accumulate as a consequence of anticancer therapies [223]. In fact, a clinical trial is testing D+Q in hematopoietic stem cell transplant survivors for these purposes (NCT02652052). In addition, two other ongoing phase II clinical trials are approved for the evaluation of the senolytic potential of D+Q on the outcomes of diabetic chronic kidney damage and Alzheimer’s disease (NCT02848131, NCT04063124). Early results from the NCT02848131 clinical trial have demonstrated that the D+Q combination has successfully cleared senescent adipocytes in humans [224]. D+Q was shown to effectively clear senescent cells in several in vitro, in vivo and ex vivo in several disease models including Alzheimer’s disease, obesity and radiation-induced tissue damage [190,191,192,193,194,195] (Table 2). Finally, it is important to mention that other preclinical evidence has suggested that D+Q might not be as effective in the clearance of senescent tumor cells as for aging-related pathologies [225].

### 4.2. Navitoclax (ABT263)

Several BCL-2 pro-survival proteins that interfere with apoptotic cell death are linked to the survival of senescent cells [226]. Navitoclax (ABT263) is a selective inhibitor of the anti-apoptotic proteins BCL-2, BCL-xL and BCL-w. Oral administration of ABT263 was found to selectively kill a variety of human and mouse senescent cells induced by radiation, oncogene-overexpression and replicative exhaustion [196]. ABT263 treatment also abrogated the IR-induced SASP response in hematopoietic cells (HSCs) and bone stromal cells, thereby improving the bone marrow status contributing to the rejuvenation of bone marrow function following radiation in mice [196]. The senolytic activity of navitoclax in senescent fibroblasts was mediated through the inhibition of the antiapoptotic protein BCL-w [196]. In another study, navitoclax was found to eliminate some, but not all forms of senescent cells; more specifically, ABT263 eliminated senescent IMR90 human lung fibroblasts, human umbilical vein epithelial cells and mouse embryonic fibroblasts, but not human senescent preadipocytes [197].

Consistent with the previous findings, another study indicated that ABT263 acts as a potent senolytic agent against type II alveolar epithelial cells exposed to radiation, both in vitro and in vivo [198]. More importantly, elimination of radiation-induced senescent cells following thoracic radiation of mice ameliorated the accompanying pulmonary fibrosis [198]. Other reports have also demonstrated a potent senolytic activity of ABT263 against senescent osteoprogenitor cells, senescent human mesenchymal stromal cells, senescent cardiac myocytes, senescent pancreatic β-cells and uterine leiomyoma cells [199,200,201,202,203,204] (Table 2). Lastly, ABT263 has been proposed to be used sequentially as an adjunct to commonly used senescence-inducing anticancer therapy, such as topoisomerase and aurora kinase inhibitors, as a novel therapeutic approach for cancer treatment [145]. ABT263 effectively eliminated senescent melanoma and lung tumor cells in vitro, mediated largely by interfering with Bcl-xL function [145].

Using radiation-induced senescent WI-38 fibroblasts, high-throughput screening also identified the potential senolytic agent piperlongumine (PL), which is a natural substance isolated from a variety of species in the genus Piper and previously reported to exert anticancer effects. In this study, PL was shown to have a broad-spectrum senolytic activity highlighted by its ability to kill senescent human WI-38 fibroblasts induced into senescence by IR, telomere dysfunction, or oncogene-overexpression [206]. In addition, the combination of ABT263 and PL showed synergistic senotoxicity [206].

Although navitoclax is now an established senolytic, its hematological toxicity limits its potential utility and efforts are focused on the identification of more specific BCL-2 inhibitors and the development of senolytics with a milder adverse effects profile. This toxicity is largely based on the fact that Bcl-xL is critical for platelets function and that navitoclax has been associated with dose-limiting thrombocytopenia. Unfortunately, the role of Bcl-xL in the maintenance of senescence is well-established, particularly in senescent tumor cells [227]. Accordingly, specific Bcl-xL inhibitors, A1331852 and A1155463, were evaluated for their ability to eliminate senescent cells; these agents were found to be effective in killing senescent HUVECs and IMR90 cells, but not senescent preadipocytes [210]. Conversely, the use of Bcl-xL sparing agents, such as venetoclax, which is a selective Bcl-2 inhibitor, was not shown to have a potent senolytic activity [145].

Curcumin, a chemical substance in turmeric, has been shown to be effective in killing senescent cells. In one report, curcumin eliminated senescent intervertebral disc cells collected from patients undergoing lower spine surgery in vitro [207]. Furthermore, the curcumin analogue EF24 exhibits a potent senolytic activity against IR-induced, oncogene-induced and replicative senescent WI-38 fibroblasts [208]. Interestingly, EF24 promoted proteasomal degradation of Bcl-xL and Mcl-1, resulting in apoptosis induction selectively in senescent cells, an effect that was synergistically enhanced when combined with ABT263 [208].

### 4.3. Fisetin

Flavones are natural compounds present in a variety of fruits and vegetables. Out of many compounds examined, fisetin was most effective at reducing senescent markers in aged mice, progeroid Ercc1^−/∆^ mice and primary culture of human adipose cells [209]. Importantly, no adverse effects of fisetin have been reported, even when given at high doses [209]. Another study confirmed the selectively of fisetin against senescent cells as it induced apoptosis in senescent but not proliferating human umbilical vein endothelial cells (HUVECs) [210]. In contrast, fisetin failed to express senolytic activity against senescent IMR90 cells, a human lung fibroblast strain, or primary human preadipocytes [210]. Such benign compounds may be better candidates for translational purposes, and in fact, a new clinical trial is set for the recruitment of patients with osteoarthritis who can benefit from fisetin’s senolytic activity (NCT04210986).

### 4.4. Metformin

In further searches for senolytic agents that might be less toxic (i.e. demonstrate an improved therapeutic index), the commonly prescribed antidiabetic drug, metformin, was explored for its potential antiaging activity. Metformin effectively enhanced the viability and proliferative capacity of mouse olfactory ensheathing cells ex vivo [211]. These cells were isolated from mice chronically receiving metformin and demonstrated decreased expression of senescence-associated markers, possibly due to the attenuation of accumulating oxidative stress. Furthermore, metformin was previously shown to interfere with the NF-κB pathway, and thus, inhibited the inflammatory drive elicited by the accumulation of senescent cells [228].

### 4.5. Panobinostat

The histone deacetylase (HDAC) inhibitor panobinostat, was investigated for its potential to clear lung, head and neck cancer cells induced into senescence by cisplatin or paclitaxel in vitro [212]. Chemotherapy-induced senescent tumor cells were more vulnerable to the toxic effects of panobinostat in comparison to their proliferating counterparts [212]. Interestingly, the senolytic activity of panobinostat was also linked to its effect on Bcl-xL expression levels [212].

### 4.6. Autophagy Modulators

Chemotherapy-induced senescence is often, if not uniformly, accompanied by autophagy, a process whereby the cell degrades subcellular organelles to generate energy and metabolic intermediates necessary for its survival [229]. However, the connection between senescence and autophagy is complex and inconsistent [230]. While it has been hypothesized that autophagy might facilitate the survival of senescent cells [231], other evidence has suggested that autophagy inhibition might, in fact, induce senescence [232]. Thus, it appears feasible that modulating the molecular processes involved in autophagy could function as a senolytic strategy when the autophagy–senescence relationship is clear. In this context, the heat shock protein 90 (HSP90) contributes to the stability of the signaling pathways implicated in autophagy regulation [233]. The HSP90 inhibitor, 17-DMAG, was shown to exert a senolytic effect on human and murine cell lines induced into accelerated senescence by oxidative stress [213]. Moreover, 17-DMAG increased the lifespan and delayed aging-related features of Ercc1^−/∆^ progeroid mice [213]. Additionally, Torin 1, a known TORC1 inhibitor, eliminated murine senescent hepatocytes ex vivo [214]. The use of rapamycin, which is also an autophagy inducer, has been established as a potent inhibitor of SASP via interfering with mTOR regulation of protein synthesis [136,138]. In addition, a recent report revealed that the phytochemical, epigallocatechin gallate (EGCG), can selectively eliminate senescent 3T3-L1 preadipocytes by downregulating PI3K/Akt/mTOR and AMPK pathways, which are important signaling pathways involved in autophagy induction and regulation, and interfering with the antiapoptotic activity of Bcl-2 [215]. Finally, consistent with the other observations, interference with mTOR function also attenuated SASP [215].

The macrolide protein synthesis inhibitors azithromycin and roxithromycin (but not erythromycin) were identified as potential senolytics through drug screening [216]. Both antibiotics are approved for the treatment of bacterial infections of the respiratory tract. Azithromycin and roxithromycin were able to effectively kill MRC-5 and BJ human fibroblasts induced into senescence by DNA damage [216]. Interestingly, the senolytic effect of both drugs was attributed to the modulation of autophagic and metabolic processes in these senescent fibroblasts [216]. These results indicate that further studies are required to elucidate the relationship between senescence and autophagy in tumor cells and the possibility of utilizing autophagy modulators as potential senolytics. On the other hand, lysosomal inhibitors (agents that interfere with the completion step of autophagy) such as bafilomycin A1, facilitated the repopulation of colorectal tumor cells induced into senescence by doxorubicin rather than decreasing their viability [158].

### 4.7. Fibrates

Fibrates are widely used for the treatment of dyslipidemia. A recent report showed that in an IL-6-induced senescence model, fenofibrate, a PPARα agonist, exerted selective killing in T/C28a2 human chondrocytes [217]. Functionally, PPARα agonism resulted in reduced proteoglycan loss in cartilage explants and exerted a protective effect against cartilage degradation, indicating its potential utility for the treatment of aging-associated osteoarthritis [217].

### 4.8. Cardiac Glycosides

Cardiac glycosides are emerging as powerful senolytic agents. Using high-throughput screening, Triana-Martínez and Picallos-Rabina et al., revealed that proscillaridin A, ouabain and digoxin have potent senolytic activity [218]. The authors induced senescence in A549 lung tumor cells (and other tumor types) using a variety of chemotherapeutic agents (bleomycin, doxorubicin, etoposide, gemcitabine, and palbociclib), followed by exposure to digoxin. Digoxin effectively eliminated senescent tumor cells in a concentration-dependent fashion [218]. In parallel, digoxin was also successful in eliminating palbociclib-induced senescent SK-MEL-103 melanoma cells, along with other cardiac glycosides such as ouabain, bufalin, cinobufagin, peruvocide, digitoxin, and convallatoxin supporting the universality of their senolytic mechanism of action. It was further elucidated that digoxin, through its potent inhibition of the membrane Na^+^/K^+^ATPase pump, results in destabilization of the senescent membrane potential and intracellular acidification, which drives the cell into apoptosis. Strikingly, digoxin resulted in a significant regression of gemcitabine-treated A549 lung tumors in vivo, highly suggesting the potential importance for the use of senolytics as complementary anticancer therapy options. The same observation was reported in patient-derived xenograft tumor cells induced into senescence by doxorubicin in vivo [218].

Another recent report by Guerrero et al. showed that ouabain (a cardiac glycoside not approved for use in the US but widely used in Europe) has a broad-spectrum senolytic activity against a variety of senescent cell types [219]. Ouabin had a comparable activity to the established senolytic navitoclax against chemotherapy-induced senescent tumor cells. Ouabin was used sequentially after senescence induction by etoposide, palbociclib, barasertib, alisertib, and tozasertib, and resulted in the killing of SKHep1 and A549 senescent tumor cells [219]. Ouabin reduced the burden of senescent cells accumulating in radiated mice in vivo, highlighting its potential effect on determining the outcome of radiotherapy in patients [219].

Despite the promising senolytic potential of cardiac glycosides [218,219], it is important to note that the digoxin and ouabin concentrations used in the referenced literatures are significantly higher than the corresponding, clinically achievable plasma concentrations of each drug. Meaning, these compounds are senolytic at concentrations beyond their therapeutic windows. For example, the senolytic activity of digoxin was best shown at 0.1 µM concentration, which is at least 200-fold higher than its safe plasma concentration (therapeutic range: 1–2.5 nM) [234]. While these reports elucidate critical molecular targets for the elimination of senescent tumor cells, it is essential to emphasize that the ideal senolytic agent would need to be utilized at clinically relevant, non-toxic doses.

## 5. Conclusions

It is clear from the preclinical literature that both conventional and targeted antitumor drugs, when studied at concentrations that are relevant to their clinical pharmacokinetic profiles, promote senescence as a primary response. Less clear is whether this is also the case in the clinic, since the shrinkage of tumors is likely to be a consequence not of senescence, but of different modes of cell death such as apoptosis, necrosis and mitotic catastrophe. However, tumor cells that survive chemotherapy and radiation treatment and persist for months, years or decades, only to ultimately recover and give rise to recurrent disease, are likely to have entered into some form of senescence (although this remains to be proven). Nevertheless, the fact that it is now generally accepted that therapy-induced senescent cells have the potential to escape from the senescent arrest and re-emerge into an actively reproductive state argues against the premise that senescence is a desirable endpoint for the development of future therapeutics except for cases where newer agents would be developed that can drive the tumors into a form of senescence that is functionally irreversible. Our current understanding of the nature of therapy-induced senescence argues in support of efforts to develop drugs with senolytic properties that can eliminate the senescent tumor cell population that is likely to be one contributor to recurrent disease. The challenge here may lie in finding compounds that are selective against the senescent tumor cells but also provide a therapeutic window that can be tolerated and does not place the patient at risk for off-target toxicities (as has long been the case for cancer therapeutics).

## Figures and Tables

**Table 1:** FDA-approved anticancer therapies that induce senescence in vitro and in vivo.

Drug Class	Drug Name	Model/Cell Line	Senescence Marker	Reference
Topoisomerase poisons/inhibitors	Doxorubicin (Adriamycin)	MCF-7, MDA-MB231	p53, SA-β-gal	[35]
		H460, A549	SA-β-gal, p21^Cip1^, p16^INK4^, p53	[36]
		HCT116, HT1080	Morphology, growth arrest, SA-β-gal	[37]
		LS174T, A2780, MCF-7, patient breast cancer tissue samples	Morphology, growth arrest, SA-β-gal, p53, p16^INK4a^	[33]
		MCF7, MDA-MB-231	SA-β-gal	[38]
		HCT116, MCF7	SA-β-gal, SASP (IL-8, VEGF), p21^Cip1^, p53, low Ki67	[39]
		DU145, LNCaP	Morphology, growth arrest, polyploidy	[40]
		K562	SA-β-gal, SAHF	[41]
		Rat-derived BMSCs and ADSCs	SA-β-gal	[42]
		MDFs, HCA2, BJ, in vivo mouse model (p16-3MR)	SA-β-gal, p21^Cip1^, p16^INK4a^, SASP (IL-1α, IL-6, Mmp-3, Mmp-9, Cxcl-1, Cxcl-10 and Ccl20), reduced Lamin B1	[28]
		SH-SY-5Y	p21^Cip1^, low Ki67, growth arrest, SA-β-gal	[43]
		HCT116	p21^Cip1^, low Ki67, growth arrest, SA-β-gal, increased granularity, morphology, SASP (IL-8), γH2AX	[43]
		MDA-MB-231	p21^Cip1^, growth arrest, SA-β-gal, morphology, SASP (IL-6, IL-8, VEGF), γH2AX	[43]
		MCF-7	p21^Cip1^, low Ki67, growth arrest, SA-β-gal, increased granularity, morphology, γH2AX	[43]
		A549	p21^Cip1^, low Ki67, growth arrest, SA-β-gal, increased granularity, morphology, SASP (IL-6, IL-8), γH2AX	[43]
	Daunorubicin	Jurkat cells	SA-β-gal, growth arrest	[44]
	Etoposide	HepG2, U2OS	SA-β-gal, p53, p21^Cip1^	[45]
		IMR-90, MEFs, BJ	SA-β-gal, growth arrest, p16^INK4^, p21^Cip1^, p53	[46]
		BJ, MEFs, B16F10	SA-β-gal, SASP (IL-6, IL-8, IL-1β)	[47]
		NRK-52E	Morphology, SA-β-gal, growth arrest, p53, p21^Cip1^	[48]
		Follicular lymphoma 3D model	SA-β-gal	[49]
	Mitoxantrone	Epithelial cells in human prostate cancer patients’ biopsies	SASP, SA-β-gal	[50]
		A549, WI38	Growth arrest, SA-β-gal, yH2AX, morphology	[51]
	Camptothecin	HCT116	SA-β-gal, morphology, SAHF, reduced BrdU incorporation	[52]
		HCT116, RKO	SA-β-gal, morphology	[53]
		HeLa, MCF7	SA-β-gal, morphology	[54]
		MNA, STA-NB-10, CLB-Ma mouse xenograft (MYCN-amplified neuroblastoma)	Reduced DNA synthesis, morphology, SA-β-gal, growth arrest, p21^Cip1^	[55]
	Irinotecan	SGC-7901, MKN-45	SA-β-gal	[56]
		A549, HCT116	p21^Cip1^, low Ki67, growth arrest, SA-β-gal, increased granularity, morphology, SASP (IL-8), γH2AX	
		SH-SY-5Y	p21^Cip1^, low Ki67, growth arrest, SA-β-gal, γH2AX	
		MDA-MB-231	p21^Cip1^, low Ki67, growth arrest, SA-β-gal, morphology, SASP (IL-6, IL-8, VEGF), γH2AX	
		MCF-7	p21^Cip1^, low Ki67, growth arrest, SA-β-gal, increased granularity, morphology, γH2AX	
	Topotecan	MNA, STA-NB-10, CLB-Ma mouse xenograft (MYCN-amplified neuroblastoma)	Reduced DNA synthesis, morphology, SA-β-gal, growth arrest, p21^Cip1^	[55]
Alkylating agents	Busulfan	Rat-derived BMSCs and ADSCs	SA-β-gal	[42]
		WI38	Growth arrest, SA-β-gal	[57]
		U2OS, MG63	SA-β-gal	[58]
		WI38	SA-β-gal	[59]
		Murine hematopoietic cells	SA-β-gal, p16^INK4^, p19^INK4^	[60]
	Temozolomide	Patient derived glioma cells	Cell cycle arrest, polyploidy, morphology	[61]
		GL261	SAHF (H3K9Me3), p53, Rb	[62]
		LN229	SA-β-gal, cell cycle arrest, SASP (IL-6, IL-8)	[63]
		In vivo (p16-3MR) mouse model	p16^INK4^	[28]
	Carmustine	GL261	SAHF (H3K9Me3), p53, Rb	[62]
	Dacarbazine	A375, B16F10	SASP	[64]
	Cyclophosphamide	HSC-bcl2 lymphoma	SA-β-gal, p53, p16^INK4^	[65]
	Melphalan	Multiple myeloma mouse model	SA-β-gal	[66]
	Mitomycin C	A549	Growth arrest, SA-β-gal, yH2AX, morphology	[67]
Platinum-based	Cisplatin	A375, B16F10, B16F10 xenografts	SASP, SA-β-gal	[64]
		A2780	SAHF (HP1-γ), morphology, SA-β-gal	[68]
		CNE1	Growth arrest, morphology, SA-β-gal	[30]
		SKOV3, TOV-21G	Morphology, SA-β-gal	[69]
		HepG2, SMMC-7721	SA-β-gal, p53, p21^Cip1^, p16^INK4^	[70]
		Follicular lymphoma 3D model	SA-β-gal	[49]
		In vivo mouse model (p16-3MR)	p16^INK4^	[28]
	Carboplatin	H1299, patients’ lung tumor samples	Cell cycle arrest, SA-β-gal, p16^INK4^, RB, downregulation of cyclin B1 and cyclin D1	[71]
	Oxaliplatin	PROb, CT26	SA-β-gal	[72]
		HepG2, SMMC-7721, patients’ colorectal tumor samples	SA-β-gal	[73]
Antimetabolites	Methotrexate	C85	p53	[74]
		C85	SA-β-gal	[75]
		MCF-7	SA-β-gal	[76]
		Rat-derived BMSCs and ADSCs	SA-β-gal	[42]
		A549	p21^Cip1^, low Ki67, growth arrest, SA-β-gal, increased granularity, morphology, SASP (IL-6, IL-8), γH2AX	[43]
		SH-SY-5Y	p21^Cip1^, growth arrest, SA-β-gal, γH2AX	[43]
		HCT116	p21^Cip1^, low Ki67, growth arrest, SA-β-gal, increased granularity, morphology, SASP (IL-8), γH2AX	[43]
		MDA-MB-231	p21^Cip1^, growth arrest, SA-β-gal, morphology, SASP (IL-6, IL-8, VEGF)	[43]
		MCF-7	p21^Cip1^, low Ki67, growth arrest, SA-β-gal, increased granularity, morphology, γH2AX	[43]
	Pemetrexed	H1650, A549, H2228, H292, H226 and H1650, A549 xenografts	SA-β-gal, morphology, SASP (IL-6, IL-8, IL-1β and MCP-1)	[77]
		A549	SASP, SA-β-gal	[78]
	Gemcitabine	Miapaca-2 and Panc-1	SA-β-gal	[79]
		AsPc1, Panc1	SA-β-gal	[80]
	Azacitidine	U2OS, MCF7	SA-β-gal, p53, growth arrest	[81]
		TPC-1	SA-β-gal	[82]
		KKU100, HuCCA1, RMCCA1	Morphology, SA-β-gal	[83]
		DU145, LNCaP	Morphology, growth arrest, polyploidy	[40]
	Bromodeoxyuridine	MNA, STA-NB-10, CLB-Ma mouse xenograft (MYCN-amplified neuroblastoma)	Reduced DNA synthesis, morphology, SA-β-gal, growth arrest, p21^Cip1^	[55]
		KKU100, HuCCA1, RMCCA1	Morphology, SA-β-gal	[83]
	5-Fluorouracil	SMMC-7721	SA-β-gal	[84]
		MDA-MB-231	SA-β-gal	[85]
	Mycophenolic acid	K562	SA-β-gal	[86]
	Hydroxyurea	STA-NB-9, STA-NB-10 MYCN amplified neuroblastoma	Morphology, increased granularity, telomere length, SA-β-gal	[87]
		MNA, STA-NB-10, CLB- primary neuroblastoma cells, mouse xenograft model for MYCN-amplified NB	Reduced DNA synthesis, morphology, SA-β-gal, cell cycle arrest, p21^Cip1^, DNA double-strand breaks	[55]
	Actinomycin D	HDF-2, NHF-3	SA-β-gal, P53, p21^Cip1^, p16^INK4^	[88]
Microtubule inhibitors/poisons	Paclitaxel	Human mesenchymal stem cells	Growth inhibition, SA-β-gal, yH2AX, morphology, SASP	[67]
		MCF-7, MEFs	Growth arrest, morphology, SA-β-gal	[89]
		A549	p21^Cip1^, low Ki67, growth arrest, SA-β-gal, increased granularity, SASP (IL-6, IL-8), γH2AX	[43]
		SH-SY-5Y	p21^Cip1^, low Ki67, growth arrest, SA-β-gal	[43]
		HCT116	p21^Cip1^, low Ki67, growth arrest, SA-β-gal, increased granularity, SASP (IL-8), γH2AX	[43]
		MDA-MB-231	p21^Cip1^, low Ki67, growth arrest, SASP (IL-6, IL-8, VEGF), γH2AX	[43]
		MCF-7	p21^Cip1^, low Ki67, growth arrest, SA-β-gal, increased granularity	[43]
	Docetaxel	DU145, LNCaP	Morphology, growth arrest, polyploidy	[40]
		PTEN null prostate tumors	SASP	[90]
	Vincristine	MCF-7	Morphology, senescence-associated lysosomal changes	[91]
	Vinblastine	Patient derived glioma cells	Cell cycle arrest and nuclear morphometric changes	[61]
Hormonal therapy	Tamoxifen	MCF-7, HCT116	SA-β-gal, p53, p21^Cip1^	[92]
			SA-β-gal, p21^Cip1^	[93]
	Fulvestrant	MCF-7, T-47D	SA-β-gal	[94]
		MCF7	SA-β-gal, morphology	[95]
	Androgen Deprivation (CSS, antiandrogen, and/or castration)	LNCaP, LAPC4	Growth arrest, p53 and p16^INK4^, SA-β-gal, low Ki67, cell cycle arrest	[96]
		LNCaP ICR castrated mice	SA-β-gal, p27^Kip1^ and p53, p21^Cip1^, SASP (IL-6 and IL-8)	[97]
		LNCaP, LAPC4	SA-β-gal, SAHF, Ki67, growth arrest, morphology, SASP,	[98]
		LNCaP, LuCaP, xenografts Patient samples	SA-β-gal, decreased proliferation, increased cellular size, p27^Kip1^, HP1γ, low Ki67	[99]
Kinase inhibitors	Imatinib	K562	SA-β-gal, growth arrest, p21^Cip1^, p27^Kip1^	[100]
	Nilotinib	H1975	SA-β-gal	[101]
	Trametinib	DMBC11, DMBC12, DMBC21, DMBC28, DMBC17	SA-β-gal	[102]
		H2030, H460, A549, MSK-LX68 patient-derived xenografts	SA-β-gal, SASP	[103]
		A549, H460, H1944, H2030, H358, H441, H2009, HCC441	SA-β-gal, growth arrest, p53, p21^Cip1^	[104]
	Vemurafenib	DMBC11, DMBC12, DMBC21, DMBC28, DMBC17	SA-β-gal	[102]
		MM034, MM070, MM074, SKMEL-28, MM050	Growth arrest, morphology, SA-β-gal	[105]
		SK-MEL-28, Mel2a, M19-Mel, SK-MEL-28, UACC-62, UACC-257, and FM88, M14, Malme 3M, Mel2a, SK-MEL-mouse xenografts	SAHF (H3K9me3), p16^INK4^, morphology, SA-β-gal, low Ki67, Rb	[106]
	Dasatinib	H1666, Cal12T	Growth arrest, reduced BrdU incorporation, SA-β-gal	[107]
		A549, H1666 H661, Cal12T	SA-β-gal, γH2AX	[108]
	Lapatinib	HCC1419, SKBR3, EFM-192A, MDA-MB-361	SA-β-gal, p15^INK4^, p16^INK4^	[109]
	Neratinib	HCC1419, SKBR3, EFM-192A, MDA-MB-361	SA-β-gal, p15^INK4^, p16^INK4^	[109]
	Afatinib	HCC1419, SKBR3, EFM-192A, MDA-MB-361	SA-β-gal, p15^INK4^, p16^INK4^	[109]
	Gefitinib	PC-9, EBC-2/R	Growth arrest, p53, p16^INK4^, p21^Cip1^, p27^Kip1^.	[110]
	Erlotinib	A549, A549 mouse xenografts	SA-β-gal, morphology	[111]
	Sorafenib	Huh7 mouse xenografts	SA-β-gal	[112]
mTOR inhibitors	Rapamycin (Sirolimus)	SMMC-7721	SA-β-gal	[84]
		HUVECs	SA-β-gal, morphology	[113]
Monoclonal antibodies	Rituximab	EHEB, RC-K8, and SD-1	Morphology, SA-β-gal	[114]
		Follicular lymphoma 3D model	SA-β-gal	[49]
	Obinutuzumab	Follicular lymphoma 3D model	SA-β-gal	[49]
	Pertuzumab	SK-BR-3	SA-β-gal, p15^INK4^, p16^INK4^	[115]
	Trastuzumab	SK-BR-3	SA-β-gal, p15^INK4^, p16^INK4^	[115]
	Bevacizumab	MIP101, RKO, SW620, SW480, MIP101 mouse xenografts	SA-β-gal	[116]
	Ranibizumab	Primary porcine retinal pigment epithelial cells	SA-β-gal, cathepsin D, amyloid β	[117]
CDK 4/6 inhibitors	Palbociclib	U87MG, U138MG, Hs683, H4, A172, LN18, LN229, CCF-STTG1, T98G, DBTRG-05MG, DKMG, GAMG, SNB19, AM38, NMC-G1, KG-1-C, U87MG and GBM39 xenograft	SA-β-gal, morphology, growth arrest	[118]
		HEK293, HeLa, U2OS	SA-β-gal, Rb, downregulated cyclin D1	[119]
		LS8817, LS141, LS0082	SA-β-gal, p53, p16^INK4^, Rb downregulated cyclin A.	[120]
		1205Lu, 983B, 983BR	SA-β-gal, SASP (IL6, IL8, CXCL1), SAHF, DNA damage	[121]
		B16-F1, B16-F10, NL212, NL216, TRIA	SA-β-gal, growth arrest, *γ*H2AX and 53BP1, p16^INK4^, p65, p21^Cip1^, p53	[122]
		SK-MEL-103, NCI-H226, Huh7, SAOS-2, UT-SCC-42B	SA-β-gal, p21^Cip1^, Rb	[123]
		AGS, MKN-45	SA-β-gal	[124]
		MCF7	SA-β-gal, γH2AX, p21^Cip1^, morphology, reduced Ki67	[125]
		Huh7, skHep1, Huh7 mouse xenografts	SA-β-gal, morphology	[112]
		Lung sections of Cdk4-deficient mice	SA-β-gal, γH2AX	[126]
		Mouse-derived sarcoma cells/tissues	53BP1, SA-β-gal, Rb	[127]
	Abemaciclib	MCF7	SA-β-gal, SAHF	[128]
	Ribociclib	Hey1	SA-β-gal	[129]
PARP inhibitors	Olaparib	HCT116	Growth arrest, morphology, SA-β-gal, γH2AX	[130]
		OV1369 (R2), OV90, OV4453, OV1946, MDA-MB-231	Growth arrest, γH2AX, 53BP1, SA-β-gal, p21^Cip1^, p27^Kip1^, p15^INK4^, p16 ^INK4^, p57, SASP (IL8)	[131]
	Niraparib	HCT116	Growth arrest, morphology, SA-β-gal, γH2AX	[130]
	Rucaparib	PC3, LNCaP, DU145, VCaP	SA-β-gal	[132]
Proteasome inhibitors	Bortezomib	U87, T98	SA-β-gal, morphology	[133]

The table summarizes frequently used, FDA-approved antineoplastic agents that have been reported to induce preclinical and clinical Therapy-Induced Senescence (TIS). Table indicates the experimental model, marker(s) used to establish senescence induction and references to corresponding research article. Key: SA-β-gal: Senescence-Associated β-galactosidase; SAHF: Senescence-Associated Heterochromatin Foci; yH2AX: Gamma-H2AX; SASP: Senescence-Associated Secretory Phenotype; HP1γ: chromatin-regulating heterochromatin protein 1γ; VEGF: Vascular endothelial growth factor. Cell lines: BMSC: Bone marrow-derived mesenchymal stem cells; AMSC: Adipose-derived mesenchymal stem cells; MDF: Mouse dermal fibroblast; MEF: Mouse embryonic fibroblast; NSCLC: Non-small-cell lung carcinoma; HAI: Hepatic Artery Chemo-Infusion; HCC: Hepatocellular carcinoma; HUVEC: Human umbilical vein endothelial cell; MALC: Multicellular Aggregates of Lymphoma Cells; GBM: Glioblastoma multiforme; HGSOC: High-grade serous ovarian cancer.

**Table 2:** Summary of preclinical evidence on the available senolytic therapies.

Senolytic	Model/Cell Line	Reference
Dasatinib + Quercetin	- Senescent HUVEC, senescent preadipocytes in vitro - Senescent MEFs, senescent bone marrow-derived murine mesenchymal stem cells in vivo - SA-β-gal positive muscle and fat tissue of irradiated single mouse limb - Progeroid Ercc1(−/∆) mice	[186]
	- Senescent lung fibroblasts and epithelial cells in bleomycin-induced lung injury/idiopathic pulmonary fibrosis mouse model	[18]
	- Senescent alveolar epithelial type (AT)II ex vivo in bleomycin-induced lung injury/idiopathic pulmonary fibrosis mouse model.	[187]
	- Senescent medial aortal cells of aging mice and hypercholesterolemia (atherosclerosis) mouse models	[188]
	- Senescent hepatocytes of dietary hepatic steatosis mouse model	[189]
	- Radiation-induced senescent preadipocytes in vivo - Senescent cells in freshly isolated human omental adipose tissue of obese individuals ex vivo	[190]
	- Arteriovenous fistula-chronic kidney disease mouse model	[191]
	- 20-month-old, transgenic tau^NFT^-Mapt^0/0^ mice	[192]
	- Aβ plaque-associated senescent oligodendrocyte progenitor cells in vivo - ZsGreen/APPPS1 p16^INK4^ reporter Alzheimer’s disease mouse model - Radiation-induced senescent N2a cells	[193]
	- Uterine fibrosis mouse model	[194]
	- Telomere dysfunction-induced senescent osteoblasts and osteocytes	[195]
Navitoclax (ABT263)	- Radiation-induced, replication-exhausted and Ras-induced senescent WI38 fibroblasts in vitro - Radiation-induced senescent human IMR90 fibroblasts, human renal epithelial cells and mouse embryonic fibroblasts in vitro - Radiation-induced senescence in p16-3MR transgenic mouse model	[196]
	- Radiation-induced senescent human umbilical vein epithelial cells, IMR90 human lung fibroblasts and mouse embryonic fibroblasts	[197]
	- Radiation-induced senescent type II alveolar epithelial cells in vitro and in vivo	[198]
	- Senescent pancreatic tissue of i4F mouse model	[199]
	- Replicative-exhausted human mesenchymal stromal cells	[200]
	- Aging-induced senescent cardiac myocytes - Myocardial infarction mouse model	[201]
	- Senescent murine pancreatic β-cells in vitro and in vivo	[202]
	- Aging mouse bone marrow stromal cells	[203]
	- WIPI1 and SLITKR4 overexpression-induced senescent uterine leiomyoma spheroid model ex vivo	[204]
Piperlongumine	- Radiation-induced senescent astrocytes in vivo - Radiation-induced cognitive dysfunction mouse model	[205]
	- SMARCB1 downregulation-induced senescent A375 melanoma cells - Therapy-induced A549 or H358 lung cancer cells	[145]
	- Radiation-induced, replication exhausted and Ras- induced senescent WI38 fibroblasts	[206]
Curcumin	- Patient-derived senescent intervertebral disc cells	[207]
	- Radiation-induced, oncogene-induced and replication-exhausted senescent WI38 fibroblasts	[208]
Fisetin	- Replication-exhausted senescent Ercc1−/− MEFs - Therapy-induced senescent IMR90 senescent cells - Progeroid Ercc1^−/∆^ mice and aged C57BL/6 mouse models - Murine and human-derived senescent adipose tissue	[209]
	- Senescent human umbilical vein endothelial cells	[210]
Metformin	- Murine olfactory ensheathing cells ex vivo	[211]
Panobinostat	- Therapy-induced senescent A549 lung and FaDu head and neck cancer cells	[212]
17-DMAG	- Oxidative-stress-induce primary Ercc1^−/−^ - progeroid Ercc1 −/∆ mouse model	[213]
Torin 1	- Murine senescent hepatocytes ex vivo	[214]
Epigallocatechin gallate (EGCG)	- Senescent 3T3-L1 preadipocytes	[215]
Bafilomycin A1	- Therapy-induced HCT116 colorectal cancer cells	[158]
Azithromycin and roxithromycin	- Therapy-induced senescent MRC-5 and BJ human fibroblasts	[216]
Fenofibrate	- Senescent T/C28a2 human chondrocytes	[217]
Cardiac glycosides	- Therapy-induced senescent A549 lung cancer cells and SK-MEL-103 melanoma cells in vitro and in vivo	[218,219]

Several natural and synthetic compounds have been tested for their senescence-eliminating effects in a variety of disease models. The table summarizes the primary current preclinical evidence demonstrating the senolytic agent and the experimental model used.
